# A8 MATERNAL IBD-ASSOCIATED MICROBIOTA SHAPES IMMUNE SYSTEM IN OFFSPRING

**DOI:** 10.1093/jcag/gwaf042.008

**Published:** 2026-02-13

**Authors:** X Mas Orea, A Hann, B Bowerman, C Bellissimo, B Pazoki, K Kennedy, M Constante, D Sloboda, H Galipeau, E F Verdu

**Affiliations:** Medecine, McMaster University Faculty of Health Sciences, Hamilton, ON, Canada; McMaster University, Hamilton, ON, Canada; Medecine, McMaster University Faculty of Health Sciences, Hamilton, ON, Canada; Medecine, McMaster University Faculty of Health Sciences, Hamilton, ON, Canada; Medecine, McMaster University Faculty of Health Sciences, Hamilton, ON, Canada; Medecine, McMaster University Faculty of Health Sciences, Hamilton, ON, Canada; Medecine, McMaster University Faculty of Health Sciences, Hamilton, ON, Canada; Medecine, McMaster University Faculty of Health Sciences, Hamilton, ON, Canada; Medecine, McMaster University Faculty of Health Sciences, Hamilton, ON, Canada; Medecine, McMaster University Faculty of Health Sciences, Hamilton, ON, Canada

## Abstract

**Background:**

Maternal microbes influence offspring immune development via placental metabolite transfer and perinatal colonization. While early-life effects are known, long-term impacts of pathogenic maternal microbiota remain unclear. Infants born to parents with prior CD diagnosis show a higher IBD risk than those whose parents developed CD later. IBD microbiota may display high proteolytic activity (HPA) that disrupts the mucus barrier and promotes inflammation. We hypothesize that such HPA microbiota imprint offspring with lasting susceptibility to colitis.

**Aims:**

1) To investigate maternal intestinal permeability in mice colonized with low PA healthy control or HPA IBD patient microbiota

2) To determine microbial metabolite exposure in offspring pre-weaning (3 wo)

3) To assess colonic and spleen immune profiles in offspring pre-weaning (3 wo)

**Methods:**

**Mouse colonization:** 6-week-old C57BL/6 germ-free (GF) mice were colonized with fecal slurries from female healthy controls (HC) or ulcerative colitis (UC) patients, and breeding pairs established.

**Permeability:** In vivo permeability was assessed in dams before pregnancy by oral gavage of 100 µL water containing 10 mg each of 4 kDa FITC– and 40 kDa TRITC–dextran. Blood was collected 1 h later with K3-Minivette for fluorescence quantification.

**Breeding:** Breeding was synchronized by co-housing males and females for 4 h (5–9 pm). Delivery was monitored at 19.5 d post-mating to ensure consistent pregnancy duration and accurate pup age. Offspring were analyzed at day 18.5 (pre-weaning).

**LPS quantification:** Plasma LPS levels were measured with the Pierce Chromogenic Endotoxin Kit (Thermo Fisher) in dams and 3-week-old offspring.

**Immune cell analysis:** Spleens were dissociated through 70 µm strainers, red cells lysed with ACK, and colon/cecum cells isolated after collagenase digestion and Percoll separation. Immune populations were profiled by Aurora 5L spectral flow cytometry.

**Results:**

Our preliminary findings demonstrate that colonization of dams prior to pregnancy with HPA IBD microbiota leads to barrier dysfunction. The high proteolytic phenotype is transferred through maternal colonization to offspring until at least 3 wo, and is associated with elevated plasma LPS and significant immune alterations. The latter are characterized by an increase CD4+ T cell frequency in the spleen and colon/cecum expressing activation and proinflammatory markers, and a blunting of innate immune cells.

**Conclusions:**

Our results suggest that maternal IBD microbiota with HPA influence offspring immune system in a sustained manner and could be a mechanism explaining increased colitis susceptibility in longitudinal cohorts. Ongoing experiments focus on broader microbial metabolite characterization, additional post natal time points and experimental colitis induction.

**Funding Agencies:**

CIHRFarncombe Family Digestive Health Research Institute

